# Excision of the Tongue

**Published:** 1858-07

**Authors:** 


### ARTICLE XIX.

### *Excision of the Tongue.*

On the 8th inst., at noon, there was great excitement exhibited in the surgical ward and operating theatre of the Royal Infirmary, Edinburg, resulting from the expectation of a very formidable surgical operation taking place that morning. The patient had, for a long period, suffered from cancer of the tongue, and Professor Syme had determined upon removing the organ bodily. Shortly after twelve o'clock, the man was led into the theatre, placed upon the table, and quickly rendered powerless through the influence of chloroform. Mr. Syme commenced by making a vertical incision through the integument covering the chin, and then sawed through the lower jaw at the symphysis. The division being made, he next proceeded to cut away the tongue at the very root, close to the hyoid bone. The arteries were quickly tied, and the hemorrhage was comparatively little, the man having lost only a few ounces of blood. The jaw was again placed together, and the integument sewed up. The patient was able to walk out of the room.

At the close of the operation, Professor Syme remarked, that the removal of the tongue bodily had been successfully performed in Italy, but the *modus operandi* was of a different nature, the incisions having been made entirely in the throat; but he considered that that mode was attended with more danger than the one he had chosen to adopt. The patient has continued well ever since, being fed with a tube, He can now, however, swallow, and a few days after the operation he spoke, or, rather breathed out the word "milk." He is cheerful, and gives every hope of recovery.

[*Lancet, Dec.* 19, 1857.

